# Perioperative fluid therapy impairs lymphatic pump function in male rats

**DOI:** 10.14814/phy2.70389

**Published:** 2025-06-10

**Authors:** Rebecca C. Harlow‐Adamek, Reetu Singh, Randolph H. Stewart, Cristine L. Heaps, Glen A. Laine, Charles S. Cox, Ranjeet M. Dongaonkar

**Affiliations:** ^1^ Michael E. DeBakey Institute for Comparative Cardiovascular Science and Biomedical Devices, Department of Veterinary Physiology & Pharmacology Texas A&M University College Station Texas USA; ^2^ Department of Pediatric Surgery The University of Texas Medical School Houston Texas USA

**Keywords:** enhanced recovery after surgery, goal‐directed fluid therapy, gut edema, intestinal dysfunction, resuscitation

## Abstract

Because of its life‐saving benefits, perioperative IV fluid therapy remains a cornerstone of medical treatment. However, it also induces sustained edemagenic stress. The resulting persistent interstitial edema—excessive fluid accumulation in the interstitium—significantly delays recovery and worsens patient outcomes. Therefore, to gain a detailed understanding of the lymphatic functional consequences of perioperative fluid therapy, this study aimed to test the hypothesis that perioperative IV fluid therapy compromises lymphatic pump function within 3 days after major surgery. Following a midline laparotomy, animals received IV fluid therapy over 48 h during recovery (FLTP). Three days post‐surgery, mesenteric lymphatic vessels from FLTP and sham surgery (CTRL) animals were isolated, and lymphatic pump function was assessed in vitro. The transmural pressure‐pump flow and circumferential length‐wall tension relationships of FLTP vessels were altered—contraction frequency and normalized pump flow and active and passive wall tensions were significantly lower than CTRL. In vessels from another group of animals with surgically produced mesenteric venous hypertension to induce sustained edemagenic stress, only the pressure‐pump flow relationship was altered similarly to FLTP. These results demonstrate the detrimental effects of perioperative fluid therapy on lymphatic pumping, which is essential for restoring interstitial fluid pressure and resolving edema and inflammation.

## 1 INTRODUCTION

### 1.1 Perioperative fluid therapy induces edemagenic stress, leading to persistent interstitial edema

The primary goal of perioperative intravenous (IV) fluid therapy is to restore and maintain intravascular volume, ensuring optimal tissue perfusion and oxygen delivery (Malbrain et al., 2020; Miller & Myles, 2019; Navarro et al., 2015; Voldby & Brandstrup, 2016; Woodcock & Michel, 2021). It is one of the most widely administered therapies, deemed indispensable for patients undergoing major surgical procedures. However, while addressing intravascular needs, this therapeutic intervention introduces edemagenic stress, resulting in persistent interstitial edema—excessive fluid accumulation in the interstitium (Chappell et al., 2008; Malbrain et al., 2020; Navarro et al., 2015; Voldby & Brandstrup, 2016; Woodcock & Michel, 2021). Because the microvascular barrier is permeable to constituents (water, electrolytes, and small‐sized solutes), crystalloid solutions, commonly used in IV administration, distribute freely between intravascular and interstitial compartments (Chappell et al., 2008; Malbrain et al., 2020; Voldby & Brandstrup, 2016; Woodcock & Michel, 2021). Additionally, the administration of crystalloid solutions decreases plasma protein concentration, reducing plasma colloid osmotic pressure (Chappell et al., 2008; Malbrain et al., 2020; Voldby & Brandstrup, 2016; Woodcock & Michel, 2021). The resulting decrease in the colloid osmotic pressure gradient across the microvascular barrier substantially increases microvascular fluid filtration rates, exacerbating edemagenic stress. Consequently, most of the administered volume accumulates in the interstitial compartment, leading to severe interstitial edema (Chappell et al., 2008; Voldby & Brandstrup, 2016; Woodcock & Michel, 2021). With clinically effective approaches to hasten edema resolution lacking, edema, left to resolve on its own, persists long after fluid therapy cessation. Nonetheless, persistent interstitial edema and the associated morbidity significantly delay recovery and worsen patient outcomes (Chappell et al., 2008; Malbrain et al., 2020; Miller et al., 2021; Miller & Myles, 2019; Navarro et al., 2015; Voldby & Brandstrup, 2016). Therefore, effectively hastening the resolution of edema is crucial for realizing optimal recovery in major surgery patients.

### 1.2 Lymphatic vessels actively drain interstitial fluid, limiting interstitial fluid accumulation

Historically, it was commonly assumed that fluid filtered from the arterial side of the microvasculature into the surrounding interstitial spaces and then was reabsorbed back into the microvasculature on the venous side (Levick & Michel, 2010; Michel et al., 2020; Woodcock & Michel, 2021). As a result, blood vessels alone were assumed to regulate interstitial fluid volume. It is currently well understood that, normally, fluid filters across the entire microvasculature into the interstitium, and reabsorption of interstitial fluid into the microvasculature is transient and highly limited (Dongaonkar et al., 2009; Levick & Michel, 2010; Michel et al., 2020; Moore Jr. & Bertram, 2018; Woodcock & Michel, 2021). Interstitial fluid in almost all organs and tissues is subsequently removed via lymphatic drainage. Interstitial fluid transuding from the surface of the organs lined with serosa is removed via lymphatic drainage of the encompassing cavity. Lymphatic pumping, extrinsically by the surrounding tissues and intrinsically by the rhythmic spontaneous contractions of lymphatic vessels, actively drains interstitial fluid and propels it through the network of coalescing collecting lymphatic vessels (Aukland & Reed, 1993; Drake et al., 1991; Gashev & Zawieja, 2001; McHale & Thornbury, 1986; Quick et al., 2007; Wiig & Swartz, 2012). Ultimately, lymph flows into the venous system via the thoracic or right lymphatic duct, returning interstitial fluid to the bloodstream. As a result, the regulation of interstitial fluid pressure and volume arises from a balance between blood and lymphatic vessel functions (Dongaonkar et al., 2008, 2009; Levick & Michel, 2010; Michel et al., 2020; Woodcock & Michel, 2021). Normally, when microvascular fluid filtration increases, lymphatic pumping and active lymph flow rates increase within minutes, limiting the excess accumulation of interstitial fluid (Aukland & Reed, 1993; Dongaonkar et al., 2009; Levick & Michel, 2010; Michel et al., 2020; Mortimer & Levick, 2004; Wiig & Swartz, 2012). Impaired lymphatic pump function, on the other hand, significantly delays edema resolution (Aukland & Reed, 1993; Dongaonkar et al., 2009; Mortimer & Levick, 2004). However, despite understanding their contribution to interstitial homeostasis, lymphatic vessels remain underexplored in mechanistic IV fluid therapy research.

### 1.3 Acute stimuli modulate lymphatic pumping, while sustained stimuli induce adaptation

Extensive investigations, both in vitro and in vivo, have demonstrated that lymphatic vessels are highly sensitive to their environment. Acute perturbations in mechanical factors such as transmural pressure and luminal flow (shear stress), as well as biochemical factors like vasoactive substances and inflammatory mediators, modulate lymphatic pumping (Zawieja et al., 2008). Furthermore, when exposed to sustained perturbations, lymphatic vessels adapt within as little as 3 days, leading to persistent changes in pumping (Dongaonkar et al., 2013, 2015; Quick et al., 2014). For example, vessels exposed to higher transmural pressures respond acutely with increased contraction frequency and adapt within 3 days to become better pumps than those exposed to lower pressures (Dongaonkar et al., 2013). Lymphatic vessels are inevitably exposed to edemagenic stress, and unsurprisingly, lymphatic pumping is modulated by consequent acute and chronic increases in microvascular fluid filtration. Acute edemagenic stress induced by IV saline infusion increased the pumping of rodent mesenteric lymphatic vessels within minutes and decreased pumping in a subset of the vessels studied (Benoit, 1991; Benoit et al., 1989; Rahbar et al., 2014). When exposed to sustained edemagenic stress induced by venous hypertension, bovine mesenteric lymphatic vessels adapted to become weaker pumps within 3 days (Dongaonkar et al., 2015; Quick et al., 2014). Unique responses influenced by the duration of edemagenic stress exposure, the source of edemagenic stress, or animal model species may have contributed to the apparent differences between rodent and bovine mesenteric lymphatic responses. Nevertheless, lymphatic adaptations that decrease active lymph flow and delay edema resolution can significantly hamper optimal patient recovery. Therefore, to gain a detailed understanding of the lymphatic functional consequences of perioperative fluid therapy, this study aimed to evaluate our hypothesis that perioperative IV fluid therapy impairs lymphatic pump function within 3 days after major surgery.

## 2 METHODS

### 2.1 Animals and treatment groups

All experimental procedures and animal care complied with animal use protocols approved by the Texas A&M University Institutional Animal Care and Use Committee. Male Sprague Dawley rats (300–380 g) were acquired (Envigo) and acclimated to the new environment before being used in experiments. Animals were maintained on a 12‐h light–dark cycle and given ad libitum access to standard chow (Inotiv, Teklad Rodent Diet 8604) and water throughout the study. Animals were randomly assigned to one of three groups: sham surgery (CTRL), surgery plus fluid therapy (FLTP), or surgery plus superior mesenteric venous hypertension (SMVH) for use in experiments to characterize the lymphatic transmural pressure‐pump flow relationship and circumferential length‐wall tension relationship.

### 2.2 Sustained exposure to edemagenic stress

#### 2.2.1 Surgical procedures

After recording weight, animals were anesthetized by inhaled isoflurane (4% for induction, 2%–3% for maintenance). Approximately 5 min prior to the incision, Lidocaine (10 mg/kg) and Bupivacaine (6 mg/kg) were administered subcutaneously in the abdomen for local analgesia. Buprenorphine SR (0.7–1 mg/kg) was administered subcutaneously in the flank for extended pain management. Animals were placed on a heated pad and prepared for aseptic surgery. Breathing pattern, heart rate, and pedal reflex were monitored throughout the surgery, and anesthesia was adjusted accordingly.

In FLTP animals, a midline incision was made after ensuring deep anesthesia with a toe pinch. The superior mesenteric vein (SMV) immediately distal to the splenic branch of the portal vein was located using wet cotton‐tipped applicators. A ~5 mm long segment of the SMV (distant from the superior mesenteric artery) was identified and manipulated to mimic isolation from the surrounding connective tissue by blunt dissection. However, the SMV was neither isolated nor ligated. Following irrigation of the cavity with sterile lactated Ringer's solution (LRS), the abdominal muscle was closed with a 4–0 nylon suture, and the skin wound was closed with a 5–0 nylon suture in a running subcuticular pattern, with care taken to bury the knots. The right jugular vein was then catheterized for fluid administration. A ~10 mm long incision was made at the neck, and a segment of the right jugular vein was isolated by blunt dissection. A pre‐filled 2F polyurethane catheter was inserted through a small interscapular incision and tunneled subcutaneously to the neck incision. The catheter was inserted ~5 mm into the jugular vein and was secured with a 4–0 nylon suture. The catheter patency was confirmed, and the catheter was then locked with heparinized LRS and plugged with an 18G PinPort (Instech) sutured in place at the interscapular incision. After the neck and back wound closure, anesthesia was discontinued, and animals were recovered and returned to the holding facility.

In SMVH animals, a midline incision was made, and the SMV was located. A ~5 mm long segment of SMV was identified and gently isolated from the surrounding connective tissue by blunt dissection. The SMV was then coarcted using a 19G blunt tip luer stub adaptor to induce sustained mesenteric venous hypertension. The luer stub adaptor was placed parallel to the isolated SMV segment, a silk suture (4–0) was tied around the adaptor and the SMV, and the adaptor was gently removed. In pilot experiments following previously reported methods, this adaptor size was determined to cause the appropriate degree of SMV occlusion to increase pressure acutely in a small mesenteric vein by 8–10 mmHg within minutes (Dongaonkar et al., 2011). Following irrigation of the cavity, the abdominal wound was closed. The right jugular vein was then catheterized, and a blood sample was collected as described above. Anesthesia was discontinued, and animals were recovered and returned to the holding facility.

In CTRL animals, a midline incision was made, and the SMV was located. The SMV was identified and manipulated to mimic isolation; however, it was neither isolated nor ligated. Following irrigation of the cavity, the abdominal wound was closed. The right jugular vein was catheterized, and a blood sample was collected. Anesthesia was discontinued, and animals were recovered and returned to the holding facility. The surgical procedures involved manipulation of the SMV and catheterization of the right jugular vein in all animals to ensure consistency of surgical procedures.

#### 2.2.2 Postsurgical monitoring and care

Postsurgical animals were housed individually and closely monitored daily during the 3‐day postsurgical recovery period. Any animals exhibiting signs of distress (e.g., weight loss >10%, frequent head pressing, or uncontrolled self‐chewing) were removed from the study. In FLTP animals, warmed LRS (80 mL/kg) was administered via the jugular catheter's PinPort an hour after recovery from anesthesia on the day of the initial surgery (day 0) and again on days 1 and 2 during the light cycle. Each animal was placed in a 6‐inch × 6‐inch container lined with bedding and food to avoid tangling or tugging of the infusion line, and warmed LRS was infused over 30 min using a syringe pump. The catheter was locked with heparinized LRS, and the animal was returned to its home cage.

#### 2.2.3 Tissue collection

On postsurgical day 3, after recording weight, animals were anesthetized using isoflurane (4% for induction, 2%–3% for maintenance). Lidocaine and bupivacaine were administered. After ensuring the animal was fully anesthetized through the absence of the pedal reflex, the abdominal wound was reopened. Jejunal sections for use in histological and tissue water content analysis were identified and ligated to prevent intestinal contents from contaminating the mesentery after excision. Non‐heparinized blood samples were collected in three microhematocrit tubes via cardiac puncture, and immediately, the jejunum and associated mesentery were carefully excised. Jejunal segments for histological and tissue water content analysis were collected, and the rest of the tissues were placed in albumin physiological salt solution (APSS) chilled on ice. Animals were euthanized by exsanguination while fully anesthetized. The correct placement of the jugular venous catheter and SMV ligature was confirmed in FLTP and SMVH animals, respectively.

### 2.3 In vitro evaluation of lymphatic transmural pressure‐pump flow relationship

#### 2.3.1 Vessel isolation, cannulation, and initial equilibration

Intestines and the attached mesentery were pinned in a dissection chamber filled with APSS and maintained at 4°C. Several mesenteric lymphatic vessels (~150‐ to 200‐μm diameter, 2‐ to 4‐mm length) were collected from each animal. Each vessel was carefully isolated using a surgical microscope. A segment with one set of valves was immediately cannulated onto two thin‐walled resistance‐matched glass pipettes (70–90 μm outer diameter) mounted on a vessel bath (Living Systems Instrumentations, CH1‐SH) filled with APSS. Glass pipettes were connected to a single APSS reservoir using polyethylene tubing. The reservoir height was adjusted to set the vessel transmural pressure. A peristaltic pump continuously replaced bath APSS, ensuring complete turnover every 30 min. The cannulated vessel was visualized using an intravital microscope, and the outer diameter of the vessel was tracked and recorded at 15 Hz using a custom‐built video caliper VI (NI LabView). The transmural pressure was set to 3 cmH_2_O, and the vessel was equilibrated for 30–45 min at 37°C. Consistent with standard practices in lymphatic vessel research, the pump flow studies were continued only in vessels exhibiting spontaneous contractions (Gashev et al., 2002; Meisner et al., 2007; Nepiyushchikh et al., 2011; Zhang, Gashev, Zawieja, Lane, & Davis, 2007). Vessels that did not exhibit spontaneous contractions after equilibration were excluded from the study.

#### 2.3.2 Active and passive data collection

The transmural pressure was briefly raised to 7 cmH_2_O, the vessel length was adjusted to remove excess axial slack, and the vessel was re‐equilibrated at 3 cmH_2_O for an additional 10 min. For active data collection, the vessel was exposed to 1, 3, 5, and 7 cmH_2_O transmural pressures in random order and allowed to reach a steady state (~5–10 min) at each pressure. The vessel was equilibrated for 5 min at 3 cmH_2_O between pressure steps. Following the active data collection, the vessel was passivated by replacing the bath APSS with Ca^2+^‐free APSS for at least 30 min. Caffeine (10 mM) in Ca^2+^‐free APSS was used in the bath to deplete the intracellular calcium stores and hasten passivation. Following the confirmation of passivity with substance P (SP, 1 μM) in Ca^2+^‐free APSS, the bath was changed back to Ca^2+^‐free APSS, and the passive diameters were recorded for 1 min at each pressure step.

#### 2.3.3 Data analysis

Lymphatic vessels were considered thin‐walled and cylindrical. The distance between the cannula tips, thus, the vessel length, was maintained constant throughout the experiment. The lymphatic end‐diastolic (EDD, μm), end‐systolic (ESD, μm), and passive (PD, μm) diameters at each transmural pressure were determined using 2 min of steady‐state diameter data. Indices characterizing lymphatic transmural pressure‐pump flow relationship—contraction frequency (CF, contractions/min), normalized stroke volume (NSV = (EDD^2^–ESD^2^)/PD^2^), normalized pump flow (NPF = CF × NSV), and diastolic tone (DT = 100 × (PD–EDD)/PD) at each transmural pressure were derived.

### 2.4 In vitro evaluation of lymphatic circumferential length‐wall tension relationship

#### 2.4.1 Vessel isolation, mounting, and initial equilibration

With the mesentery pinned in a dissection chamber, lymphatic vessels were located, and surrounding connective tissue was carefully dissected away. Lymphatic vessels, being thin‐walled, collapse when isolated and unpressurized. Therefore, to minimize manipulation and vessel damage, two gold‐plated tungsten wires (25 μm diameter, 2.5 cm length) were inserted into the lumen before the vessel was entirely excised from the mesentery. The vessel segment (~2 mm long) around the two wires was then cut and transferred to a myograph chamber (DMT 320, preheated and calibrated) filled with degassed and chilled APSS. The wires were secured parallel to each other to the jaws of the myograph chamber, and any attached air bubbles were removed. The chamber was then transferred to an inverted microscope stage, and the vessel was visualized. The (axial) length of the vessel was measured using imaging software (HCImage), and wall tension (= force/(2 × axial length)) was derived from the measured force and recorded at 15 Hz throughout the experiment. The bath APSS was heated, and vessel equilibration was initiated. When bath APSS reached 37°C, the force transducer was re‐zeroed, bath APSS was replaced, and the 60‐min equilibration period continued.

#### 2.4.2 Vessel preloading

Following equilibration, the vessel was first transitioned from the initial unloaded state (with the inner edges of the tungsten wires almost touching each other) to a minimally loaded state. Two jaws were separated in 10 μm steps with ~1–2 min equilibration at each step until a stable level of wall tension was recorded for ~2 min. The tension was recorded as preload. With the vessel stretched between the two mounting wires, the internal circumferential length of the vessel (L) henceforth was calculated as = mounting wire circumference (π × wire diameter (25 μm)) + (2 × wire diameter (25 μm)) + (2 × distance between the inner edges of the wires).

#### 2.4.3 Active and passive data collection

The step resulting in preloading the vessel marked the “stretch” in the first iteration of the three‐step activation sequence—stretch, stimulation, and washout. For stimulation, bath APSS was replaced with substance P (1 μM) in K‐APSS (60 mM K^+^) to stimulate the vessel maximally, and the vessel was equilibrated until the recorded tension reached a steady state over ~5–10 min. The bath was then flushed with APSS thrice, and the vessel was equilibrated for ~5 min. The activation sequence, (1) stretch by 10 μm, (2) stimulation with SP + K‐APSS, and (3) washout, was repeated until the agonist‐stimulated developed tension (= total wall tension after stimulation – total wall tension after stretch) decreased at two successive iterations after reaching a maximal level. Next, lymphatic passive length‐tension data were collected. The vessel was passivated for 30 min by replacing bath APSS with Ca^2+^‐free APSS. Passivity was confirmed by a lack of response to substance P (1 μM) in Ca^2+^‐free APSS. The vessel was transitioned to an unloaded state by moving the jaws together until the inner edges of the mounting wires were almost touching, and the force transducer was re‐zeroed. The passive vessel was then stretched incrementally by distancing the wires in 10 μm steps, and passive tension was recorded for 1 min at each step.

#### 2.4.4 Data analysis

Agonist‐stimulated developed tension was calculated as the difference between total wall tension after stimulation and total wall tension after a stretch for each iteration of the activation sequence. The circumferential length, L, corresponding to maximal agonist‐developed tension, was defined as the optimal length indicated by L_max_. Active tension after stretch was calculated as the difference between total wall tension after stretch and passive tension determined at the corresponding L. Similarly, active tension after stimulation was calculated as the difference between total wall tension after stimulation and passive tension at the corresponding L. An exponential curve (passive wall tension $=\alpha e^{\mathrm{\beta L}/L_{\max}}$) was fitted to passive length‐tension data to characterize passive wall tension as a function of normalized circumferential length (L/L_max_) (Meisner et al., 2007). The constants, α and β, were derived using passive tensions recorded at 10 incremental stretch levels, two below L_max_, one at L_max_, and seven above L_max_. Given the rightward shift typically observed in the length‐tension relationship under passivated conditions, additional stretch levels beyond L_max_ were included in the analysis.

### 2.5 Determination of other relevant variables

#### 2.5.1 Weight change

The initial weight of an animal was recorded on day 0 at the beginning of the initial survival surgery and the final weight on day 3 at the beginning of the terminal surgery. The percent weight change of an animal was calculated as 100 × (final weight–initial weight)/initial weight.

#### 2.5.2 Hematocrit and serum protein concentration

Non‐heparinized blood samples collected via cardiac puncture on postsurgical day 3 were utilized to determine hematocrit and serum protein concentration. Hematocrit values were determined for each microhematocrit tube and then averaged across the three replicates. The serum protein concentration was promptly measured using a refractometer and averaged across the three replicates.

#### 2.5.3 Intestinal water content

Jejunal segments were flushed with APSS, gently blotted dry, and weighed (wet weight). The segments were subsequently dried in an oven at 60°C over several days until a consistent dry weight was achieved. Intestinal tissue water content was estimated by calculating the tissue wet‐to‐dry weight ratio as (wet weight–dry weight)/dry weight.

#### 2.5.4 Characterization of intestinal morphology

Jejunal segments were flushed with APSS, gently blotted dry, and immediately fixed in formalin. Subsequently, 5 μm thick cross sections of paraffin‐embedded segments were cut, processed, and stained with hematoxylin and eosin (H&E). The mucosal morphology was quantitatively assessed using the Chiu intestinal injury scoring system in a blinded fashion (Chiu et al., 1970).

### 2.6 Statistical analysis

Data from one vessel per animal were used to characterize lymphatic transmural pressure‐pump flow or the circumferential length‐tension relationship. Data are presented as mean ± SD, and *n* indicates the number of vessels used. Vessels in the CTRL (sham surgery) group animals served as controls to evaluate the effects of fluid therapy and superior mesenteric venous hypertension. Measured diameters and derived functional indices of vessels in CTRL, FLTP, and SMVH group animals were compared by two‐way repeated measures ANOVA (transmural pressure as a repeated measure; Geisser–Greenhouse correction for unequal variability of differences) followed by Tukey's test for pairwise comparisons. One‐way ANOVA with Tukey's test was used to compare total wall tensions after stretch and stimulation and agonist‐stimulated developed tensions at L = L_max_ between the CTRL, FLTP, and SMVH vessels. Active tensions after stretch and stimulation at L = L_max_ and constants α and β characterizing the shape of the passive length‐tension relationship of CTRL, FLTP, and SMVH vessels were compared by one‐way ANOVA followed by Tukey's test for pairwise comparisons.

Initial and final weights, percent weight change, hematocrit, serum protein concentrations, tissue water content, and intestinal injury score between CTRL, FLTP, and SMVH animals were compared by one‐way ANOVA followed by Tukey's test for pairwise comparisons. Data are presented as mean ± SD, and *n* indicates the number of animals. Differences were considered significant at *p* < 0.05.

### 2.7 Solutions and chemicals

Substance P and caffeine were purchased from Fisher Scientific. All other chemicals were purchased from MilliporeSigma. APSS was composed of (in mM) 145.0 NaCl, 4.7 KCl, 2.0 CaCl_2_, 1.2 MgSO_4_, 1.2 NaH_2_PO_4_, 0.02 EDTA, 5.0 glucose, 2.0 sodium pyruvate, 3.0 MOPS, and 0.5% purified bovine serum albumin (Cat# 3116964001). Ca^2+^‐free APSS was prepared by substituting CaCl_2_ with 3.0 mM EDTA. K‐APSS was prepared by equimolar substitution of NaCl with KCl to achieve 60 mM K^+^. Substance P (Cat# NC9179888) was dissolved in 14 M acetic acid and stored as 50 mM aliquots. Caffeine (Cat# ICN15011483) was dissolved in boiling water and stored as 1 M aliquots. Aliquots were mixed with APSS or Ca^2+^‐free APSS before being added to the bath.

## 3 RESULTS

Two animals (CTRL, *n* = 1; FLTP, *n* = 0; SMVH, *n* = 1) exhibited signs of distress during recovery and were consequently removed from the study.

### 3.1 Lymphatic transmural pressure‐pump flow relationship

**FIGURE 1:**
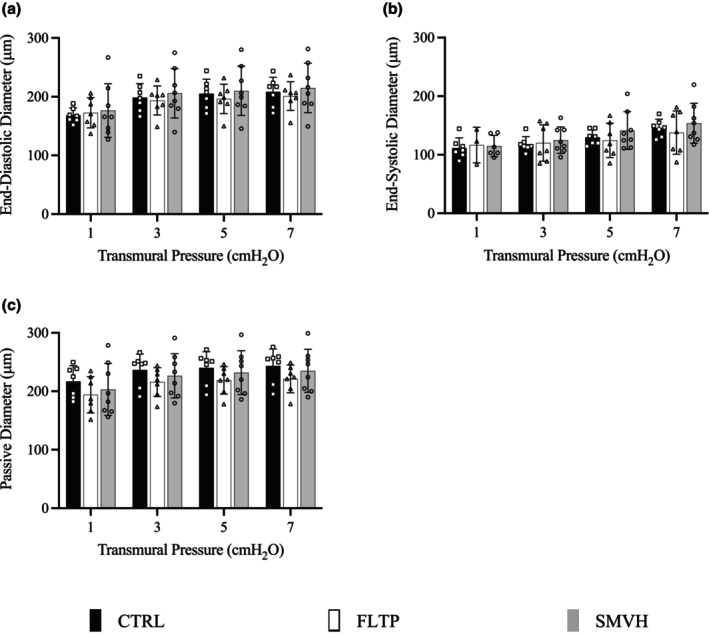
End‐diastolic (a), end‐systolic (b), and passive diameters (c) measured in mesenteric lymphatic vessels in CTRL (sham surgery, *n* = 7, filled‐black), FLTP (surgery plus fluid therapy, *n* = 7, open), and SMVH (surgery plus superior mesenteric venous hypertension, *n* = 8, filled‐gray) groups. Notably, some FLTP (*n* = 4) and SMVH (*n* = 2) vessels did not exhibit spontaneous contractions at 1 cmH_2_O transmural pressure, resulting in undefined systolic diameters for these vessels. The diameters in CTRL, FLTP, and SMVH vessels were compared using a two‐way ANOVA with transmural pressure as a repeated measure, employing Geisser–Greenhouse correction for unequal variability of differences. ANOVA showed no significant differences in end‐diastolic (*p* = 0.7971), end‐systolic (*p* = 0.5843), or passive diameters (*p* = 0.4318) among groups. “*n*” indicates the number of vessels. *p* < 0.05, a significant difference.

Figure 1 summarizes the end‐diastolic, end‐systolic, and passive diameters of CTRL (*n* = 7), FLTP (*n* = 7), and SMVH (*n* = 8) vessels at four transmural pressures. Some vessels in FLTP (*n* = 4) and SMVH (*n* = 2) did not exhibit spontaneous contractions at 1 cmH_2_O transmural pressure. Consequently, systoles were absent in these vessels, and their systolic diameters were undefined. The end‐systolic diameters of the remaining vessels in FLTP and SMVH were used for comparison. There were no significant differences in any of the diameters between the groups.

**FIGURE 2:**
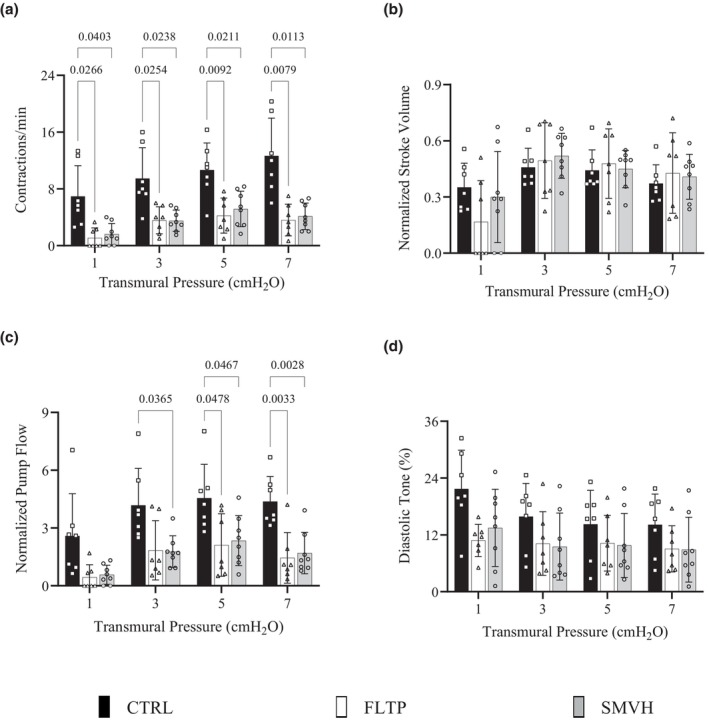
Pumping indexes of mesenteric lymphatic vessels in CTRL (sham surgery, *n* = 7, filled‐black), FLTP (surgery plus fluid therapy, *n* = 7, open), and SMVH (surgery plus superior mesenteric venous hypertension, *n* = 8, filled‐gray) groups. Contraction frequency (a), normalized stroke volume (b), normalized pump flow (c), and diastolic tone (d) are shown. Notably, some FLTP (*n* = 4) and SMVH (*n* = 2) vessels did not exhibit spontaneous contractions at 1 cmH_2_O transmural pressure, evident by zero contraction frequency, normalized stroke volume, and normalized pump flow. To compare the pumping indexes of CTRL, FLTP, and SMVH vessels, a two‐way ANOVA was performed with transmural pressure as a repeated measure and Geisser–Greenhouse correction for unequal variability of differences, followed by Tukey's test for pairwise comparisons, as appropriate. ANOVA revealed significant effects of treatment on contraction frequency (*p* = 0.0002) and normalized pump flow (*p* = 0.0019), but not on normalized stroke volume (*p* = 0.8965) or diastolic tone (*p* = 0.0589). “*n*” indicates the number of vessels. *p* < 0.05, a significant difference.

The derived lymphatic pumping indices—contraction frequency, normalized stroke volume, normalized pump flow, and diastolic tone—at four transmural pressures are depicted in Figure 2. At a transmural pressure of 1 cmH_2_O, a contraction frequency of zero reflects the absence of spontaneous contractions in FLTP and SMVH vessels. Contraction frequency (Figure 2a) in both FLTP and SMVH was significantly lower than in CTRL at all transmural pressures. There were no significant differences in contraction frequency between FLTP and SMVH at any transmural pressure. Normalized pump flow (Figure 2c), compared to the CTRL group, was significantly lower in FLTP at 5 and 7 cmH_2_O and in SMVH at 3, 5, and 7 cmH_2_O transmural pressures. There were no significant differences in normalized pump flow between FLTP and SMVH at any transmural pressure. Normalized stroke volume (Figure 2b) and the diastolic tone (Figure 2d) did not differ significantly between the three groups.

### 3.2 Lymphatic circumferential length‐wall tension relationship

**FIGURE 3:**
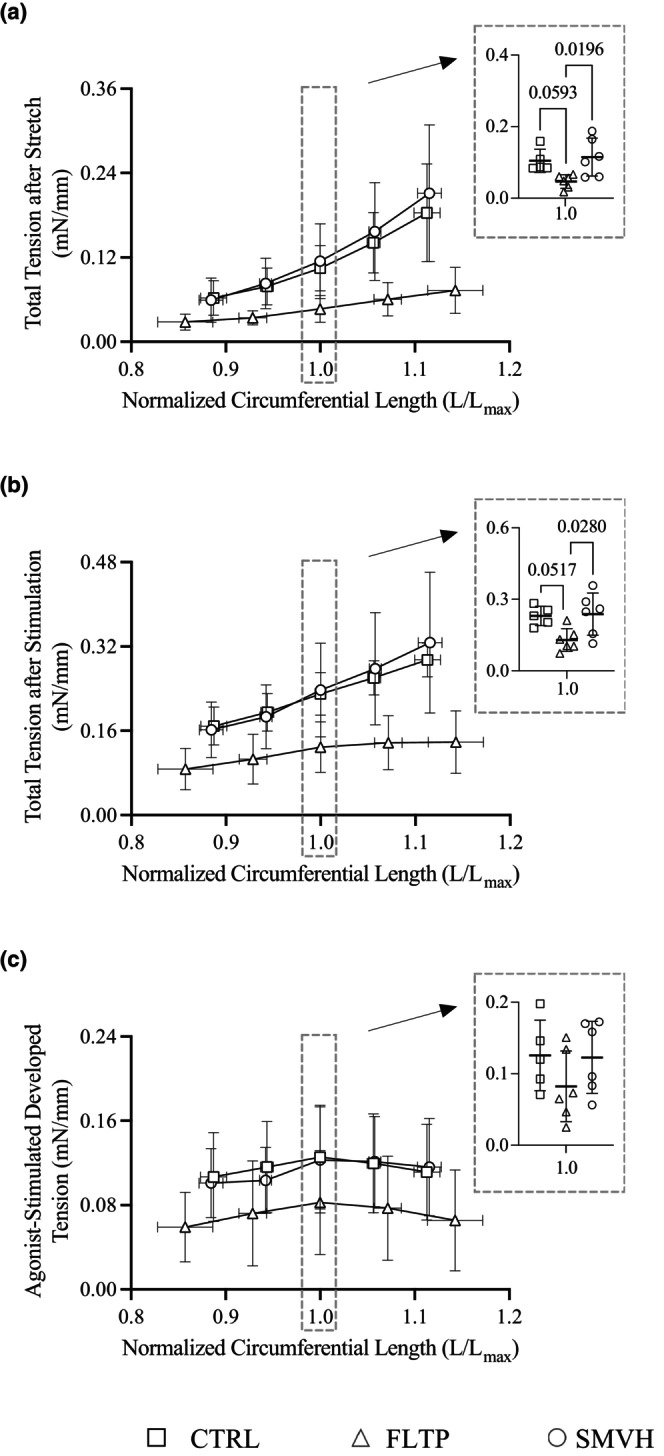
Circumferential length‐wall tension relationships of mesenteric lymphatic vessels in CTRL (sham surgery, *n* = 5, □), FLTP (surgery plus fluid therapy, *n* = 6, ∆), and SMVH (surgery plus superior mesenteric venous hypertension, *n* = 6, ○) groups. Total wall tensions after stretch (a) were subtracted from total wall tensions after stimulation (b) to determine agonist‐stimulated developed tensions (c). Total wall tensions after stretch and after stimulation and agonist‐stimulated developed tensions at L = L_max_ of CTRL, FLTP, and SMVH vessels were compared using one‐way ANOVA followed by Tukey's post hoc test. At L = L_max_, one‐way ANOVA detected significant differences in total wall tension after stretch (*p* = 0.0168) and after stimulation (*p* = 0.0202), but not in agonist‐stimulated developed tension (*p* = 0.2874). Total wall tensions after stretch and after stimulation did not differ significantly between CTRL and FLTP (*p* = 0.0593, *p* = 0.0517, respectively) or between CTRL and SMVH (*p* = 0.8980, *p* = 0.9801, respectively).' n' ndicates the number of vessels. *p* < 0.05, a significant difference.

Figure 3 depicts the circumferential length‐wall tension relationships of mesenteric lymphatic vessels in CTRL (*n* = 5), FLTP (*n* = 6), and SMVH (*n* = 6) groups. The internal circumferential length, L, was normalized to the optimal length, L_max_. Recorded total wall tensions after stretch (Figure 3a) and after stimulation (Figure 3b) and calculated agonist‐stimulated developed tensions (Figure 3c) corresponding to five iterations of activation sequence (two at L < L_max_, one at L = L_max_, and two at L > L_max_), are summarized. Compared at L = L_max_, total wall tensions after stretch and after stimulation were not significantly different in FLTP or SMVH from CTRL vessels. However, both wall tensions at L = L_max_ were significantly lower in FLTP than in SMVH vessels. There were no significant differences in agonist‐stimulated developed tensions between the three groups.

**FIGURE 4:**
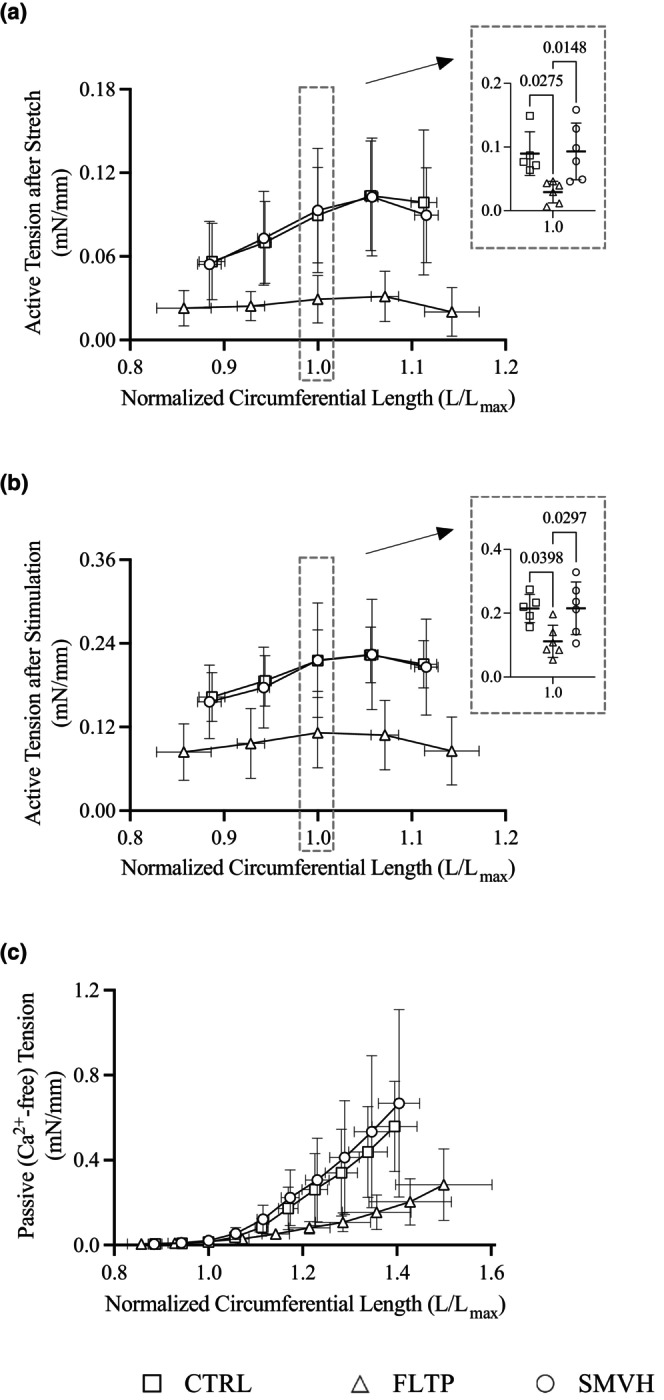
Circumferential length‐wall tension relationships of mesenteric lymphatic vessels in CTRL (sham surgery, □), FLTP (surgery plus fluid therapy, ∆), and SMVH (surgery plus superior mesenteric venous hypertension, ○) groups. Active tensions after stretch (a) and after stimulation (b) were determined by subtracting passive tensions (c) from total wall tensions after stretch and after stimulation (CTRL, *n* = 5; FLTP, *n* = 6; SMVH, *n* = 6). Data from a vessel in the FLTP group exhibiting an abrupt change in passive tension at higher stretch levels was excluded from the passive length‐tension analysis (CTRL, *n* = 5; FLTP, *n* = 5; SMVH, *n* = 6). Active tensions after stretch and after stimulation at L = L_max_, as well as the constants α and β of CTRL, FLTP, and SMVH vessels, were compared by one‐way ANOVA followed by Tukey's test for pairwise comparisons. One‐way ANOVA detected treatment effects on active tensions after stretch (*p* = 0.0098) and after stimulation (*p* = 0.0184) at L = L_max_, and constants α (*p* = 0.0116) and β (*p* = 0.0040). Active tensions after stretch (*p* = 0.9840) and after stimulation (*p* = 0.9998) did not differ significantly between CTRL and SMVH groups. The constants α and β of FLTP vessels (α, 1.02e‐4 ± 9.01e‐5; β, 5.62 ± 1.31) differed significantly from those of CTRL (α, 1.37e‐6 ± 1.19e‐6, *p* = 0.0196; β, 9.79 ± 0.85, *p* = 0.0077) and SMVH vessels (α, 7.38e‐6 ± 1.42e‐5, *p* = 0.0217; β, 9.58 ± 2.55, *p* = 0.0080). However, the two constants were not significantly different between CTRL and SMVH vessels (α, *p* = 0.9792; β, *p* = 0.9813). ‘*n*’ indicates the number of vessels. *p* < 0.05, a significant difference.

Lymphatic active tensions after stretch (Figure 4a), active tensions after stimulation (Figure 4b), and passive tensions (Figure 4c) as a function of normalized internal circumferential length are summarized in Figure 4. Active tensions after stretch and after stimulation at L = L_max_ were significantly lower in FLTP vessels than in CTRL and SMVH vessels. However, the two active tensions were not significantly different between CTRL and SMVH vessels.

No abrupt changes in passive tensions were observed across the 10 stretch levels, indicating the absence of tissue damage—except in one vessel from the FLTP group at the last three stretch levels. Consequently, the passive tension data from this vessel were excluded, and the constants, α and β, were derived using the data from the remaining five FLTP vessels (CTRL, *n* = 5; FLTP, *n* = 5; SMVH, *n* = 6). Significant differences in the passive length‐tension relationship between FLTP and the other two groups were revealed by the values of α and β, which characterize the shape of the passive length‐tension relationship. The values of α were significantly larger, and β were significantly smaller in FLTP than in CTRL and SMVH vessels. The two constants were not different between CTRL and SMVH vessels.

### 3.3 Comparison of other relevant variables

**FIGURE 5:**
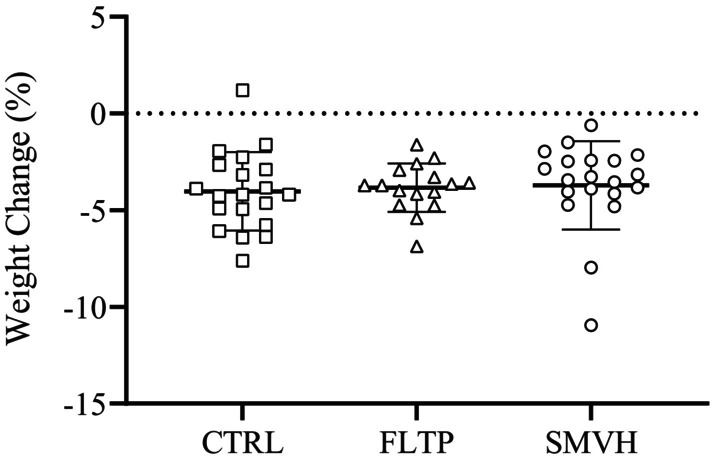
Percent change in animal weights during the 3‐day recovery period across the CTRL (sham surgery, *n* = 20, □), FLTP (surgery plus fluid therapy, *n* = 16, ∆), and SMVH (surgery plus superior mesenteric venous hypertension, *n* = 20, ○) groups. Percent weight changes, analyzed using one‐way ANOVA, did not differ significantly among the three groups (*p* = 0.8781). “*n*” indicates the number of animals. *p* < 0.05, a significant difference.

All animals experienced a decrease in weight over the 3‐day recovery period. However, there were no significant differences in initial or final weights between CTRL, FLTP, and SMVH groups. The percent weight change, the difference between initial and final weights as a percentage of the initial weight, was also not significantly different between the three groups (Figure 5). Hematocrit, serum protein concentration, and intestinal water content were not significantly different between CTRL, FLTP, and SMVH groups (Table 1).

**TABLE 1:** Hematocrit, serum protein concentration, and intestinal tissue water content (wet‐to‐dry weight ratio) determined on postsurgical day 3 in CTRL (sham surgery), FLTP (surgery plus fluid therapy), and SMVH (surgery plus superior mesenteric venous hypertension) animals. One‐way ANOVA did not detect significant differences in hematocrit (*p* = 0.5746), serum protein concentrations (*p* = 0.3289), or tissue water content (*p* = 0.4503) between CTRL, FLTP, and SMVH groups.

	Hematocrit (%)	Serum protein (g/dL)	Tissue water
CTRL	44.9 ± 1.3 (*n* = 8)	6.62 ± 0.15 (*n* = 8)	3.96 ± 0.42 (*n* = 12)
FLTP	44.5 ± 1.7 (*n* = 7)	6.74 ± 0.11 (*n* = 7)	4.00 ± 0.40 (*n* = 12)
SMVH	44.0 ± 1.3 (*n* = 9)	6.67 ± 0.17 (*n* = 10)	4.15 ± 0.33 (*n* = 13)

*Note*: Data presented as mean ± SD. ‘*n*’ indicates the number of animals. *p* < 0.05, a significant difference.

**FIGURE 6:**
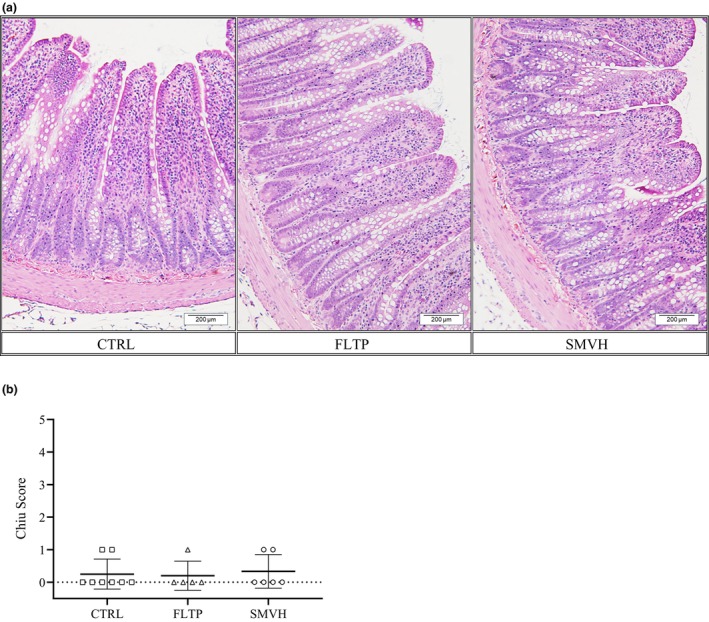
Intestinal morphology in animals from CTRL (sham surgery, *n* = 8, □), FLTP (surgery plus fluid therapy, *n* = 5, ∆), and SMVH (surgery plus superior mesenteric venous hypertension, *n* = 6, ○) groups. Formalin‐fixed, paraffin‐embedded jejunal sections stained with hematoxylin and eosin (a) were imaged at 200× magnification. Mucosal morphology was evaluated using the Chiu scoring system (b) (Chiu et al., 1970). Scores were compared across groups using one‐way ANOVA, and no significant differences were observed (*p* = 0.8947). “*n*” indicates the number of animals. *p* < 0.05, a significant difference. Scale bar: 200 μm.

Figure 6a depicts representative hematoxylin and eosin‐stained jejunal sections from animals in CTRL, FLTP, and SMVH groups. Normal mucosal architecture with intact villi was observed in the jejunum of animals in all three groups. The Chiu scores, which quantitatively characterized jejunal mucosal morphology, did not differ significantly between the three groups (Figure 6b).

## 4 DISCUSSION

In the present study, the effects of perioperative fluid therapy (FLTP) on mesenteric lymphatic pump function in a rodent model were compared to mesenteric venous hypertension (SMVH) and sham surgery controls (CTRL). These data demonstrate that IV fluid infusion impairs lymphatic pump function within 3 days. Both pressure‐pump flow and length‐wall tension relationships of FLTP vessels were altered, and their contraction frequency, normalized pump flow, and active and passive wall tensions were significantly lower than in CTRL vessels. In the case of SMVH vessels, like in FLTP vessels, the pressure‐pump flow relationships were altered, and their contraction frequency and normalized pump flow were significantly lowered compared to CTRL vessels. However, their length‐wall tension relationships were unaffected. To date, fluid therapy research has primarily focused on the elusive goal of limiting fluid therapy‐induced edemagenic stress. However, enhancing lymphatic function as a means to hasten edema resolution has yet to be thoroughly investigated and could prove crucial for optimizing the recovery of patients undergoing major surgery.

Major surgery patients receive intravenous fluids intraoperatively and during the immediate postoperative period (Chappell et al., 2008; Gupta & Gan, 2016; Malbrain et al., 2020; Miller & Myles, 2019). In most cases, fluid therapy is discontinued within 48 h of recovery (Gupta & Gan, 2016; Malbrain et al., 2020; Miller & Myles, 2019; Navarro et al., 2015). Although the use of crystalloids versus colloids for fluid therapy has been highly debated and remains controversial, in the case of major surgeries, an isotonic, balanced crystalloid, for example, Lactated Ringer's Solution (LRS), is commonly recommended (Allen, 2014; Gupta & Gan, 2016; Miller & Myles, 2019; Navarro et al., 2015). Therefore, in the present study, an LRS bolus was administered thrice, once immediately after surgery and again on days 1 and 2 during the 48‐h postsurgical period. Following IV fluid bolus infusion, hemodilution and reductions in plasma colloid osmotic pressures persist over several hours (≥8 h), and urinary fluid excretion remains significantly elevated over at least 22 h after infusion (Drummer et al., 1992; Lobo, 2004; Lobo et al., 2006). Accordingly, lymphatic responses were assessed on day 3 of recovery from surgery, ~24 h after the last IV fluid infusion. The 3‐day recovery period also aligned with the earliest documented lymphatic adaptation. Bolus infusion was preferred over continuous infusion to minimize animal handling or use of restraints and associated stress.

The goal in the present study was to induce mild‐to‐moderate levels of edemagenic stress, and the production of severe intestinal edema was not targeted. Earlier studies using a rodent gastrointestinal (GI) model involving abdominal laparotomy, a major surgical procedure, reported mild intestinal edema from 0.5 to 12 h after IV fluid administration (Cox Jr. et al., 2008; Moore‐Olufemi et al., 2009; Moore‐Olufemi, Xue, Allen, et al., 2005a; Moore‐Olufemi, Xue, Attuwaybi, et al., 2005; Radhakrishnan et al., 2009; Uray et al., 2006, 2007). In the present study, this previously established model was adapted to induce sustained edemagenic stress. The same LRS volume as used in these earlier studies was administered to FLTP animals. In SMVH animals, the increase in the mesenteric venous pressure was limited to levels previously reported to acutely elevate intestinal microvascular pressure and induce intestinal edema, without significantly impairing intestinal blood flow (Drake & Gabel, 1991; Dunbar et al., 1989; Korthuis et al., 1988). However, the lack of intestinal edema on day 3 in SMVH animals in the present study is consistent with the previous report in animals with comparable levels of venous hypertension (Quick et al., 2014). Intestinal microvascular and interstitial adaptations to counter the effects of sustained venous hypertension could have also contributed (Huang et al., 2001; Townsley et al., 1995). Therefore, the absence of elevated tissue water content in FLTP and SMVH animals compared to CTRL at the end of the 3‐day recovery period was not surprising; indeed, it corroborated the mild edemagenic stress in these animals. Conversely, both IV fluid therapy and mesenteric venous hypertension, which induced mild edemagenic stress, proved potent enough to trigger lymphatic dysfunction.

To avoid the confounding effects of an uncontrolled environment in vivo, changes in lymphatic pump function were evaluated in vitro under control conditions. This approach also eliminated the acute effects of LRS infusion and mesenteric venous hypertension on lymphatic transmural pressures and luminal flow rates in vivo (Benoit, 1991; Benoit et al., 1989; Dunbar et al., 1989; Rahbar et al., 2014; Stewart & Laine, 2001). Lymphatic transmural pressure‐pump flow relationships, characterized in vitro under isobaric conditions, allowed estimation of active pump flow. Lymphatic circumferential length‐wall tension relationships, examined using wire myography under isometric conditions, enabled the evaluation of lymphatic muscle function with maximal activation (Davis et al., 2008; Zhang, Gashev, Zawieja, & Davis, 2007; Zhang, Gashev, Zawieja, Lane, & Davis, 2007). These complementary approaches yielded a comprehensive understanding of how lymphatic vessels adapt to sustained edemagenic stress and facilitated the disentangling of the differing responses to IV fluid therapy and venous hypertension. Mesenteric lymphatic vessels from sham‐operated (CTRL) animals used for comparison enabled the characterization of lymphatic responses to edemagenic stress while controlling for the confounding effects of surgical stress. Additionally, in vitro experiments conducted under controlled conditions confirmed that the observed persistent effects were due to adaptation and not merely temporary acute regulatory responses.

The seminal work by Benoit et al. demonstrated that acute edemagenic stress induced in vivo by IV saline infusion enhances lymphatic pumping within minutes (Benoit et al., 1989). Combined with several hypothesized mechanisms, elevated lymphatic diastolic pressure was identified to mediate increased lymphatic pumping within minutes of initiating IV fluid infusion. Subsequent studies further corroborated the acute edemagenic stress‐induced enhanced lymphatic pumping; although, in a subset of the vessels studied, lymphatic pumping was decreased (Benoit, 1991; Rahbar et al., 2014). However, the direct comparison of increased lymphatic pumping observed in vivo in these earlier studies with lower lymphatic pumping in FLTP and SMVH than in CTRL vessels in vitro in the present study is complicated by the fact that the former is an acute regulation of lymphatic pumping by edemagenic stress, and the latter is a lymphatic adaptation to sustained edemagenic stress. Lymphatic acute regulatory responses characterized in vivo while the vessels were exposed to edemagenic stress, in fact, are comparable with lymphatic transmural pressure‐pump flow relationships characterized in vitro while the vessels were exposed to different transmural pressures in the present study. Consistent with the Benoit study, increased lymphatic contraction frequency and pump flow at higher transmural pressures were observed in all vessels (FLTP, SMVH, and CTRL) in the present study. Indeed, these responses of FLTP and SMVH vessels to transmural pressure suggest that the acute regulatory mechanisms were preserved in FLTP and SMVH vessels. The evident differences in the pressure‐pump flow relationships of FLTP or SMVH vessels compared to CTRL vessels, therefore, reveal the lymphatic adaptation responses to sustained edemagenic stress. These adaptive responses of FLTP and SMVH vessels are consistent with the previous reports in bovine mesenteric lymphatic vessels exposed to sustained edemagenic stress (Quick et al., 2014).

Only a small number of lymphatic wire myography studies have been published, but the recorded length‐tension relationships in the present study are consistent with earlier reports (Dougherty et al., 2008, 2014; Gashev et al., 2012; Nepiyushchikh et al., 2011; Telinius et al., 2017; Zhang, Gashev, Zawieja, & Davis, 2007; Zhang, Gashev, Zawieja, Lane, & Davis, 2007). The length‐wall tension curves after stretch and after stimulation and length‐agonist‐stimulated developed tension curves of CTRL and SMVH vessels were similar to those reported earlier in wild‐type rat mesenteric lymphatic vessels (Dougherty et al., 2008; Zhang, Gashev, Zawieja, & Davis, 2007). The optimal preload corresponding to the maximal agonist‐stimulated developed tension, that is, the total wall tension after stretch at L = L_max_, reported earlier in wild‐type rat mesenteric lymphatic vessels was comparable to those in CTRL and SMVH vessels (Dougherty et al., 2008; Zhang, Gashev, Zawieja, & Davis, 2007). The maximal agonist‐stimulated developed tensions in wild‐type rat mesenteric lymphatic vessels reported earlier were also comparable to those in CTRL and SMVH vessels (Dougherty et al., 2008; Zhang, Gashev, Zawieja, & Davis, 2007). The differences in experimental treatments (CTRL and SMVH vs. wild type) and lower K^+^ concentrations used for stimulation in the present study (60 mM vs. 145 mM used earlier) probably contributed to the lower agonist‐stimulated developed tensions in CTRL and SMVH than in wild‐type vessels. The optimal preload and maximal agonist‐stimulated developed tension of FLTP vessels were lower than those of CTRL, SMVH, and wild‐type vessels (Dougherty et al., 2008; Zhang, Gashev, Zawieja, & Davis, 2007). The differences in optimal preload observed between the treatment groups caution against assuming a universal optimum preload. Rather, they underscore the need to experimentally determine optimal preload, particularly in cases of in vivo intervention where adaptation is plausible.

Characterizing wall tensions in passivated vessels has seldom been reported in earlier wire myography studies. Passive length‐wall tension relationships were significantly different in FLTP vessels compared to CTRL and SMVH vessels, revealing that fluid therapy‐induced sustained edemagenic stress affects lymphatic passive properties. Furthermore, at L = L_max_, the active tensions obtained as the difference between passive tensions and wall tensions after stretch and stimulation were significantly lower in FLTP than in CTRL and SMVH vessels. These data suggest (1) increased compliance (decreased tissue elastic modulus) of FLTP vessels and (2) a decreased ability of muscle cells in FLTP vessels to generate tension in response to elevated preload (contractility) or stimulation (excitability). Active and passive length‐tension relationships of SMVH vessels were not significantly different from those of CTRL vessels. Earlier bovine studies reported that active tensions in response to KCl stimulation were significantly lower in vessels exposed to sustained mesenteric venous hypertension than those in sham‐treated vessels (Dongaonkar et al., 2015). However, different—isovolumetric versus isometric—experimental approaches employed in the earlier bovine and present rodent studies limit the comparison of lymphatic responses. The passive length‐wall tension relationships, minimally confounded by the utilized approaches, were not significantly different in vessels exposed to sustained mesenteric venous hypertension than those in sham‐treated vessels in either study.

The differences between CTRL and FLTP or SMVH vessels in lymphatic pumping at a transmural pressure primarily resulted from decreased contraction frequency in FLTP and SMVH vessels. Both intravenous crystalloid infusion and mesenteric venous hypertension increase microvascular fluid filtration, thus increasing intestinal interstitial fluid pressure, which acts as lymphatic inlet pressure. When lymphatic inlet pressure rises above lymphatic outlet pressure, lymph flows passively, driven by the inlet‐to‐outlet axial pressure gradient (Gashev et al., 2002; Quick et al., 2007, 2009). In response to the increased shear stress, lymphatic vessels transition to conduits, reducing the active lymph flow (Gashev et al., 2002; Quick et al., 2007, 2009). Decreased pumping in FLTP and SMVH vessels is consistent with these studies, suggesting the possible effects of increased lymph flow and endothelial shear stress on contraction frequency.

Earlier studies demonstrated that comparable IV fluid therapy or mesenteric venous hypertension does not initiate intestinal inflammatory responses (Cox Jr. et al., 2008; Moore‐Olufemi et al., 2009; Moore‐Olufemi, Xue, Allen, et al., 2005a; Moore‐Olufemi, Xue, Attuwaybi, et al., 2005; Radhakrishnan et al., 2009; Uray et al., 2006, 2007). Consistent with these reports, the jejunal epithelial and mucosal morphology and the Chiu scores were not significantly different between CTRL, FLTP, and SMVH groups in the present study. It is possible that the inflammatory signaling molecules originating in the mesentery surrounding the vessels contributed to responses in FLTP and SMVH vessels. Numerous inflammatory mediators, including prostaglandins, bradykinin, histamine, and substance P, have been demonstrated to affect lymphatic contractile behavior (Amerini et al., 2004; Chakraborty et al., 2011; Dobbins et al., 1990; Fox & von der Weid, 2002; Hashimoto et al., 1994; Johnston, 1987; Wu et al., 2006). The differences in active responses of FLTP and SMVH vessels suggest the possible role of more than one pathway, including nitric oxide (NO) and prostacyclin (PGI_2_) signaling, reported to mediate the effects of elevated endothelial shear stress on lymphatic peacemaking (Davis, 2018; Davis & Zawieja, 2025). The altered passive responses observed in FLTP vessels alone further indicate the potential involvement of additional pathways specifically influenced by sustained edemagenic stress induced by fluid therapy, but not by that induced by mesenteric venous hypertension. With the lymphatic consequences of fluid therapy now established, revisiting these complex and resource‐intensive experiments is justified in future studies to systematically investigate the key variables and mechanisms underlying lymphatic responses and functional recovery.

Decades of extensive clinical research have highlighted the detrimental consequences of overly liberal or overly restrictive fluid regimens (Malbrain et al., 2020; Messina et al., 2021; Miller et al., 2021; Miller & Myles, 2019; Navarro et al., 2015; Thacker et al., 2016). Consequently, current guidelines advocate restricting administered fluid volume and maintaining an overall positive fluid balance within specified limits (Kehlet, 2020; Malbrain et al., 2020; Navarro et al., 2015; Thacker et al., 2016). While this approach has been correlated with a reduced incidence of severe morbidity compared to both overly liberal and overly restrictive fluid management strategies, it does not entirely eliminate edemagenic stress and the development of interstitial edema and associated morbidity (Miller et al., 2021; Thacker et al., 2016). Furthermore, regardless of the volume or type of fluid administered, IV fluid therapy is compounded by the effects of increased microvascular permeability in major surgery patients and is thus bound to induce sustained edemagenic stress (Chappell et al., 2008; Codner et al., 2016; Helander et al., 2019). Lymphatic adaptation observed in the FLTP vessels (with decreased contraction frequency, decreased developed tension, and increased compliance) would enable the vessels to dilate maximally and decrease resistance to lymph flow, enhancing passive lymph flow (Quick et al., 2007, 2009). Such lymphatic adaptation would increase the clearance of interstitial fluid and would be beneficial when fluids are administered intravenously; however, with impaired lymphatic pumping, it would delay edema resolution after stopping fluid therapy (Jamalian et al., 2017; Quick et al., 2007, 2009). Impaired lymphatic pumping and reduced lymph flow would also promote the interstitial accumulation of immune cells, inducing inflammation and delaying the resolution of inflammation in inflamed tissues. Therefore, focusing on lymphatic function to effectively hasten edema resolution is crucial for optimal recovery of major surgery patients.

Observed diminished lymphatic pump function following IV fluid therapy over 48 h in the present study underscores the critical need to explore the role of the lymphatic system in trauma patient care. While large‐volume IV fluid administration is typically discouraged, trauma patients often necessitate aggressive resuscitative measures to stabilize their hemodynamics (Balogh et al., 2002, 2003; Moore‐Olufemi, Xue, Allen, et al., 2005b; Radhakrishnan et al., 2006; Shah et al., 2011). However, the resulting extreme edemagenic stress leads to severe intestinal edema, subsequently raising intra‐abdominal pressure, culminating in secondary abdominal compartment syndrome (ACS), and necessitating decompressive laparotomy (Balogh et al., 2002, 2003; Moore‐Olufemi, Xue, Allen, et al., 2005b; Radhakrishnan et al., 2006; Shah et al., 2011). Following decompression, the enhanced venous return from the lower body reduces the need for continued crystalloid resuscitation. However, even after discontinuing fluid administration, severe intestinal edema persists, preventing abdominal wall closure for days or even weeks in extreme cases (Balogh et al., 2002, 2003; Moore‐Olufemi, Xue, Allen, et al., 2005b; Radhakrishnan et al., 2006; Shah et al., 2011). Given that lymphatic pumping is necessary for resolving edema, reversing edemagenic stress‐induced lymphatic pump failure holds promise in hastening edema resolution, enabling earlier abdominal wall closure and substantially improving survival prospects.

## FUNDING INFORMATION

The authors declare that no funding was received for the research, authorship, and/or publication of this article.

## CONFLICT OF INTEREST STATEMENT

The authors declare that there is no conflict of interest regarding the publication of this article.

## ETHICS STATEMENT

All experimental procedures and animal care complied with animal use protocols approved by the Texas A&M University Institutional Animal Care and Use Committee.

## Data Availability

The data that support the findings of this study are available from the corresponding author upon reasonable request.
